# Feasibility and safety of the distal radial access for vascular access interventional therapy

**DOI:** 10.1007/s12928-025-01127-4

**Published:** 2025-05-04

**Authors:** Koji Kuroda, Ayaka Murakami, Takafumi Todoroki, Masamichi Iwasaki, Junichi Imanishi, Souichiro Yamashita, Wataru Fujimoto, Makoto Takemoto, Masanori Okuda

**Affiliations:** Department of Cardiology, Hyogo Prefectural Awaji Medical Center, 1-1-137 Shioya, Sumoto, Hyogo 656-0021 Japan

**Keywords:** Clinical research, Vascular access intervention therapy, Distal radial artery access

## Abstract

Distal radial access (DRA) has emerged as an alternative approach to reduce the risk of radial artery occlusion in coronary angiography. Vascular access intervention therapy (VAIVT) is traditionally used to treat arteriovenous fistula stenosis. However, the feasibility and safety of DRA for VAIVT have not been established. This study aimed to evaluate the feasibility and safety of using DRA for VAIVT. We included 421 consecutive VAIVT procedures for upper limb arteriovenous fistulas retrospectively. DRA for VAIVT was performed in 181 procedures (DRA group), and the remaining procedures were approached through the brachial artery or vein (SA group: standard access group). Clinical follow-up was performed to evaluate the incidence of hematoma and dialysis access-associated steal syndrome (DASS). The mean follow-up duration following VAIVT was 24.0 months. The VAIVT success rate did not differ significantly between the groups (DRA: 97.8% vs. SA: 98.3%; *P* = 0.73), neither did the incidence of puncture site hematoma (DRA: 0.0% vs. SA: 0.8%; *P* = 0.51). There were no complications with DASS symptoms in the DRA group during the clinical follow-up. In the DRA group, 90 patients underwent a subsequent VAIVT procedure, and 85 patients (94.4%) underwent a subsequent VAIVT by DRA. DRA is a feasible and safe strategy for VAIVT and could be considered an option for VAIVT.

**Graphical abstract:**

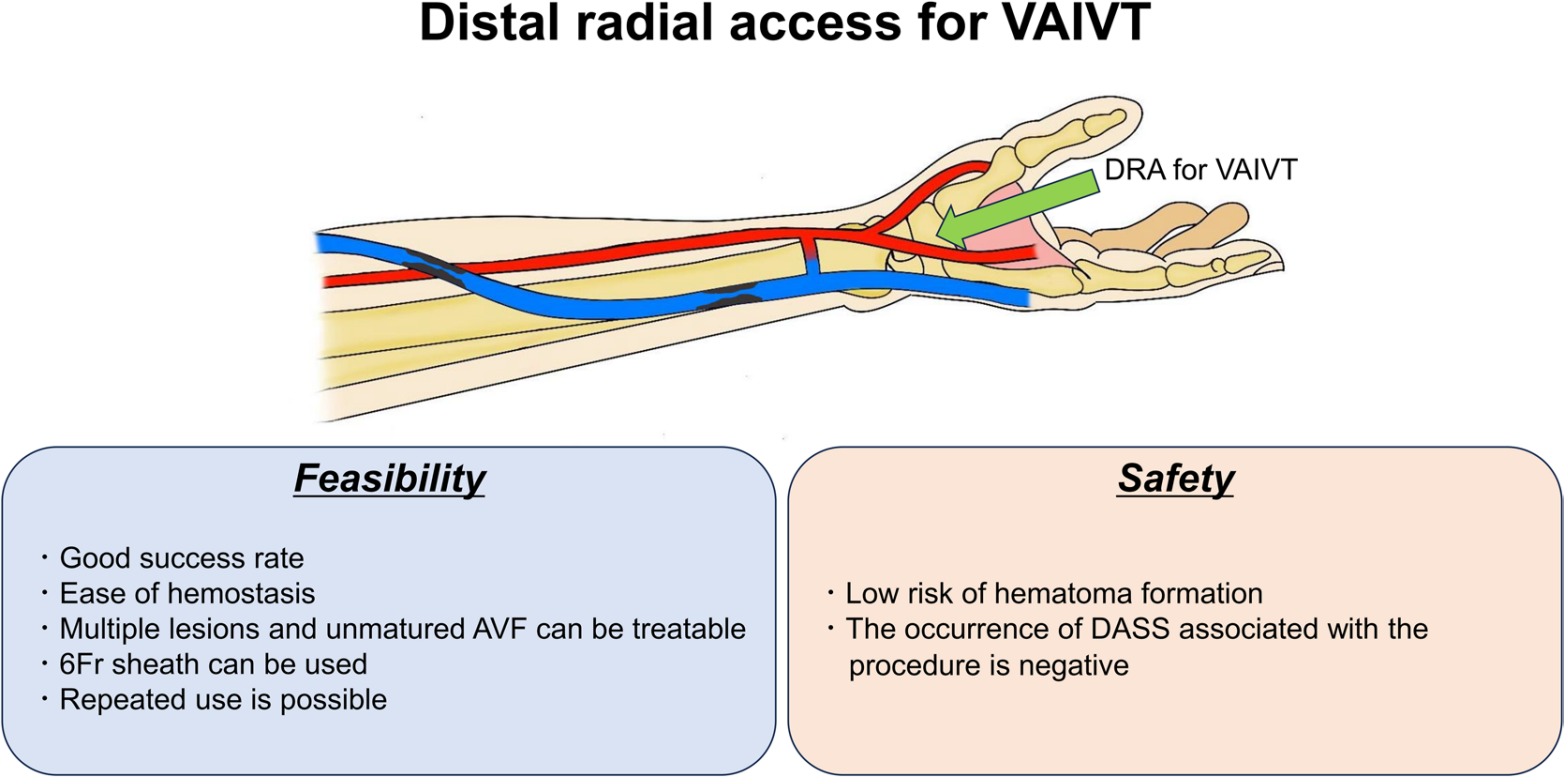

**Supplementary Information:**

The online version contains supplementary material available at 10.1007/s12928-025-01127-4.

## Introduction

Approximately, 4 million people worldwide have recently undergone renal replacement therapy [1]. According to the United States Renal Data System (USRDS), in 2022, Japan ranked third in the world after Taiwan and South Korea regarding the prevalence of dialysis patients per million in the population. The most common approach site for dialysis is through an arteriovenous fistula (AVF) constructed in the upper limb rather than a central venous dialysis catheter inserted into the internal meridian or subclavian vein due to risks such as infection [2]. While continuous hemodialysis via the AVF has been widely adopted, the incidence of dysfunction due to vascular stenosis in the AVF circuit, which results in inadequate hemodialysis, is high [3, 4]. Vascular access intervention therapy (VAIVT) has historically been used to treat AVF stenosis [5]. It usually involves either antegrade or retrograde access along the AVF circuit, depending on the lesion being treated; therefore, the access site is often selected on the brachial artery or the venous side of the AVF. However, brachial artery puncture is associated with a high risk of complications [6]. Conversely, transvenous access requires additional angiography with compression of the venous limb to visualize the juxta-anastomotic area, which can result in additional procedure time and radiation exposure [7]. The trans-radial approach (TRA) has recently been reported as an option in VAIVT for AVF [7, 8]. TRA can reduce the operator's fluoroscopic exposure and provide a more efficient contrast examination than the transvenous approach [8]. Distal radial access (DRA) through the anatomical snuff box of the hand has emerged as an alternative approach to minimize the risk of radial artery occlusion and access site hematoma in coronary angiography and percutaneous coronary intervention (PCI) procedures [9–11]. However, in VAIVT procedures, the feasibility and safety of DRA remain unclear. Therefore, this study aimed to determine the feasibility and safety of DRA for VAIVT.

## Methods

### Patient population

This was a single-center, retrospective study. From January 2020 to December 2023, consecutive VAIVT procedures performed at Awaji Medical Center were included. We excluded cases in which the AVF was constructed outside the upper limbs. Patients were categorized into two groups based on their access sites. Patients who underwent VAIVT by the distal radial artery were assigned to the DRA group, and those who underwent VAIVT by the brachial artery or venous on the AVF circuit were assigned to the SA group. Clinical information was carefully reviewed using electronic medical records from our hospital. This study was approved by the ethics committee of the Hyogo Prefectural Awaji Medical Center, and written informed consent was obtained from all enrolled patients. This study was performed per the Declaration of Helsinki guidelines and registered in the UMIN Clinical Trial Registry (UMIN 000055455).

### Outcome variables and definitions

Clinical outcome data (mean follow-up, 24.0 ± 14.3 months) were obtained by reviewing outpatient records. The primary end points were the success of VAIVT procedures (free from circuit reconstruction) and the secondary end points were complications after VAIVT. Circuit reconstruction was defined as surgical circuit reconstruction and did not include re-VAIVT. Complications were defined as hematoma or new appearance of dialysis-associated steal syndrome (DASS) after VAIVT. Hematoma was defined based on a diameter greater than 5 cm according to an easy-to-use hematoma scale [12]. These data were obtained by personnel blinded to the VAIVT procedure.

### VAIVT procedures and hemostasis

The access site was at the discretion of the operator. In cases where the operator selected the distal radial artery, it had to be palpable and have a diameter of at least 2 mm on ultrasound examination. If the ultrasound examination showed obstruction or narrowing of the vessel diameter from the anastomosis to the puncture site, the access site was changed to SA. The patient’s hand was placed on a comfortable arm support, with the wrist in passive ulnar flexion. After the subcutaneous anesthetic injection of xylocaine into the anatomical snuffbox above the distal radial artery, the artery was punctured under ultrasound guidance. After a successful anterior wall puncture, a 6 French sheath (Ultra High Flow Sheath, Medikit, Tokyo, Japan) was inserted into the distal radial artery. Subsequently, periprocedural anticoagulation was administered appropriately to all patients based on the standard hospital protocol of 60 units/kg up to 5000 international units of unfractionated heparin. After VAIVT, hemostasis of the distal radial artery was achieved using the Stepty device (NICHIBAN, Tokyo, Japan). The Stepty device was used to apply pressure during sheath removal and was protected with tape to prevent it from falling out. It was removed after 2 h of rest. If hemostasis was not achieved within 2 h, the same procedure was repeated for up to 2 h.

In cases where the operator chose the brachial artery or vein in the AVF circuit, a 6 French sheath (Ultra High Flow Sheath, Medikit, Tokyo, Japan) was inserted into the target vessel after a successful anterior wall puncture. Periprocedural anticoagulation was administered at the same dose as in the DRA procedure. After the VAIVT procedure, hemostasis was achieved using manual compression with a hemostatic aid (Chitostat, Medikit, Tokyo, Japan). After achieving hemostasis in cases of brachial artery punctures, the patients underwent overnight observation and a 6-h bed rest. In cases of venous punctures, hemostasis was confirmed using the same protocol as for DRA. In both groups, the time required to puncture was defined as the time from local anesthesia to sheath insertion. The procedure time was defined as the time from time-out to sign-out.

### Statistical analysis

Statistical analyses were conducted using SPSS software version 29 (IBM Corp., Armonk, NY, US). For continuous variables, the two groups were compared using a two-tailed, unpaired *t* test or Wilcoxon test. Discrete variables are presented as percentages, and comparisons were performed using Chi-square analysis or Fisher’s exact test.

## Results

### Patient and lesion characteristics

**Fig. 1:**
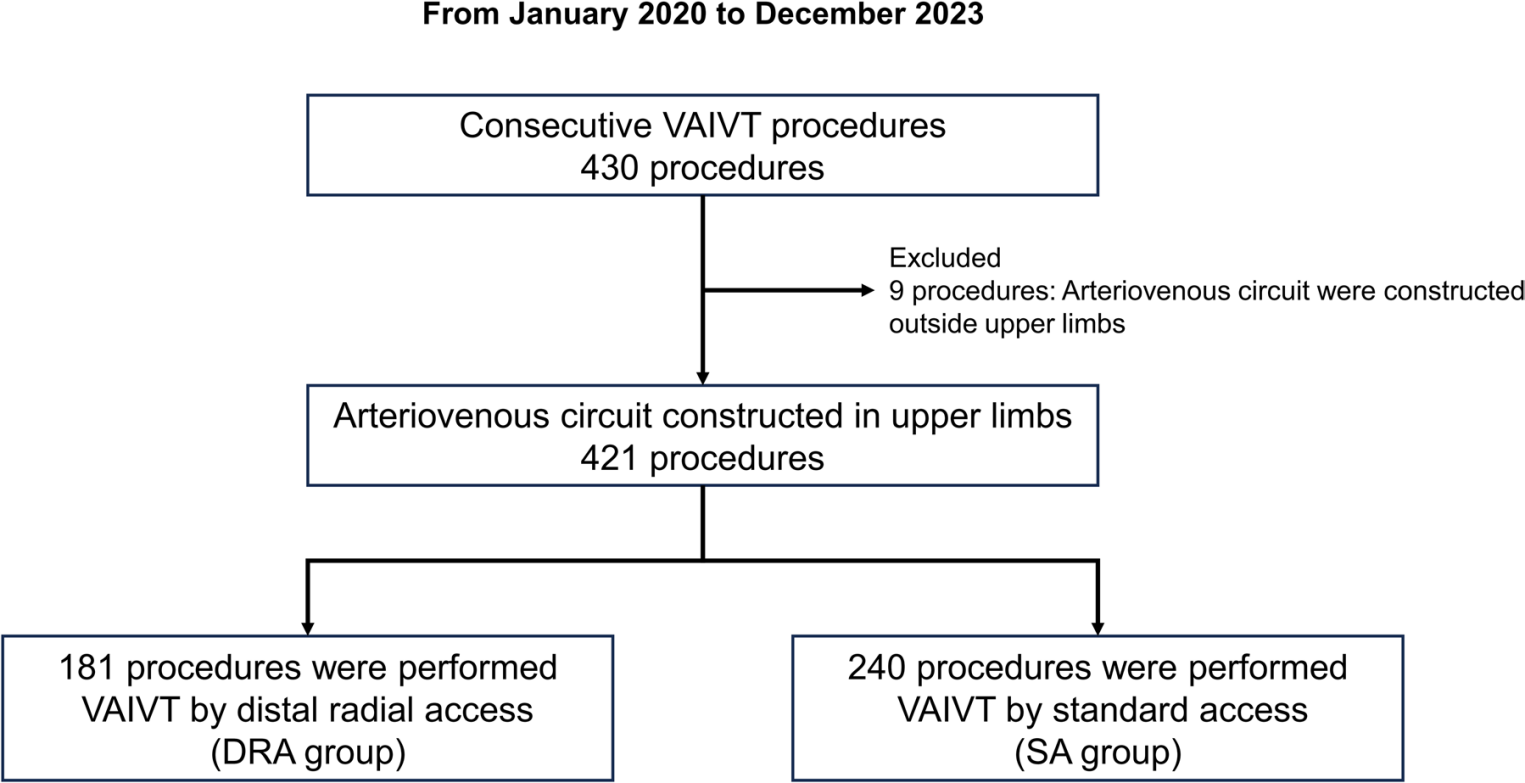
Study population

A total of 421 VAIVT procedures were included. Among them, 181 procedures were performed using VAIVT with DRA, while the remaining 240 were performed using VAIVT with SA (Fig. 1). In the SA group, 25 procedures involved the brachial artery. The groups did not significantly differ regarding baseline patient characteristics during the VAIVT procedure (Table 1). However, the percentage of early phase VAIVT (duration of treated arteriovenous [AV] circuit use at the time of VAIVT < 6 months) was significantly higher in the DRA group than in the SA group (21.5% vs. 8.8%, *P* < 0.01). Regarding the lesion and procedural characteristics, the percentage of AV grafts was significantly lower in the DRA group. In addition, the proportion of circuit sites in the upper arm was significantly smaller in the DRA group; correspondingly, the balloon diameter was significantly smaller in the DRA group. There was no significant difference between the two groups regarding stenosis or obstruction; however, stenosis with multiple lesions was significantly more significant in the DRA group. The time required for puncture was significantly longer in the DRA group than in the SA group. However, there was no significant difference between the two groups regarding the whole procedure time (Table 2).

**Table 1:** Baseline patient’s characteristics at VAIVT procedures

Variables	DRA (*n* = 181)	SA (*n* = 240)	*P* value
Age (years)	74.8 ± 10.7	72.8 ± 10.1	0.07
Duration of dialysis (months)	76.5 ± 105.5	96.5 ± 109.5	0.07
Duration of AV circuit use at the time of VAIVT (months)	46.1 ± 66.2	57.5 ± 66.0	0.08
duration of treated AV circuit use at the time of VAIVT < 6 months	39 (21.5)	21 (8.8)	< 0.01
Male	114 (63.0)	158 (65.8)	0.55
BMI (kg/m^2^)	21.9 ± 4.7	21.9 ± 4.6	0.98
Diabetes mellitus	85 (47.0)	111 (46.3)	0.89
Hypertension	141 (77.9)	197 (82.1)	0.29
Dyslipidemia	63 (34.8)	81 (33.8)	0.82
Former smoking	64 (35.4)	95 (39.6)	0.38
Current smoking	23 (12.7)	33 (13.8)	0.76
History of CAD	47 (26.0)	81 (33.8)	0.09
History of LEAD	30 (16.6)	57 (23.8)	0.07

Values are presented as mean ± SD or absolute numbers (%)

*AV circuit* arteriovenous circuit, *CAD* coronary artery disease, *LEAD* lower extremity artery disease

**Table 2:** Lesion and procedural characteristics

Variables	DRA (*n* = 181)	SA (*n* = 240)	*P* value
AV circuit			< 0.01
AVF	174 (96.1)	204 (85.0)	
AVG	7 (3.9)	36 (15.0)	
Site of the AV circuit			< 0.01
Forearm	161 (89.0)	172 (71.7)	
Upper arm	20 (11.0)	68 (28.3)	
Culprit lesion			
Stenosis	127 (70.2)	172 (71.7)	0.50
Multiple lesions	47 (26.0)	21 (8.8)	< 0.001
Occlusion	54 (29.8)	68 (28.3)	0.50
Procedures			
Balloon diameter (mm)	5.1 ± 1.0	5.4 ± 1.3	< 0.01
Use of DCB	20 (11.1)	35 (14.6)	0.29
Time required for puncture (min)	5.3 ± 4.5	2.9 ± 2.5	< 0.001
Procedure time (min)	64.6 ± 35.1	69.5 ± 36.9	0.17
Radiation exposure (mGy)	34.0 ± 65.4	37.7 ± 58.9	0.55

Values are presented as mean ± SD or absolute numbers (%)

*AV circuit* arteriovenous circuit, *AVF* arteriovenous fistulas, *AVG* arteriovenous graft, *DCB* drug-coated balloon

### Clinical outcomes

The success rate of VAIVT was not significantly different between the two groups, and all cases requiring circuit reconstruction in both groups involved occlusion (Online Resource 1). In a comparison of circuit reconstruction between the DRA and SA groups within subgroups, there were no subgroups with significant differences (Online Resource 2). Additionally, the rate of complications after VAIVT did not differ significantly between the two groups. Notably, none of the patients in the DRA group experienced DASS symptoms during clinical follow-up. Hematoma at puncture site respectively occurred in one case involving the brachial artery and venous approach. The incidence of circuit revascularization during the clinical follow-up was not significantly different between the two groups (Table 3).

**Table 3:** Clinical outcomes

Variables	DRA (*n* = 181)	SA (*n* = 240)	*P* value
Follow-up duration (months)	22.7 ± 12.9	24.9 ± 15.2	0.38
Success of VAIVT			
Free from AV circuit reconstruction	177 (97.8)	236 (98.3)	0.73
Complications			
Hematoma of puncture site	0 (0.0)	2 (0.8)	0.51
DASS	0 (0.0)	1 (0.4)	1.00
Circuit revascularization	90 (49.7)	128 (53.3)	0.38

Values are presented as mean ± SD or absolute numbers (%)

*AV circuit* arteriovenous circuit, *DASS* dialysis-associated steal syndrome

In the DRA group, 90 procedures were performed after VAIVT. Among these patients, 85 (94.4%) underwent repeated VAIVT procedures using DRA. On the other hand, five cases were performed using SA. In four cases, this was due to narrowing of the distal artery (< 2.0 mm) on echocardiography, and the incidence of distal radial artery occlusion was observed in only one case without symptoms of DASS (Table 4).

**Table 4:** Cases in the DRA group that underwent revascularization

Variables	DRA group that underwent revascularization (*n* = 90)
Access site of the next VAIVT procedures	
Distal radial artery	85 (94.4)
Brachial artery	1 (1.1)
Venous	4 (4.4)
Reason for use of standard access	
Shrinking of the distal radial artery (< 2.0 mm)	4 (4.4)
Occlusion of the distal radial artery	1 (1.1)

Values are presented in absolute numbers (%)

## Discussion

The main findings of this study are as follows. (1) The VAIVT success rates were not significantly different between DRA and SA. (2) Moreover, the rate of complications was not significantly different between DRA and SA after VAIVT. (3) No DRA patients experienced DASS symptoms during the clinical follow-up. (4) 94.4% of patients who underwent VAIVT with DRA were able to use DRA again for repeated VAIVT procedures.

A recent study demonstrated the feasibility and safety of DRA during coronary catheterization in patients undergoing hemodialysis [13]. On the other hand, few reports have been made about the distal radial approach in VAIVT. Watanabe et al. reported 12 cases of VAIVT using the distal radial artery, with a 100% success rate and no bleeding complications or radial artery occlusion [14]. Xuxin et al. reported similar results in 37 cases [15]. In this study, we clarified the feasibility and safety of DRA, including the possibility of reuse in VAIVT procedures, with a more significant number of included procedures (421 procedures, including 181 DRA procedures) and a more extended follow-up period (24.0 ± 14.3 months after VAIVT).

### Feasibility of distal radial access for VAIVT

This study showed a 97.8% success rate for the DRA group, which was not significantly different from that of the SA group. All cases requiring circuit reconstruction in both groups were occlusion cases, and the proportion of occluded lesions was approximately 30% in both groups. The success rate of VAIVT for stenotic lesions is highly favorable, while patency on the day after VAIVT was reported to be approximately 90% for obstructed lesions [16]. Taking the proportion of obstructed lesions in this study into account, we consider the success rate to be within a reasonable range. To the best of our knowledge, there have been no detailed reports specifically addressing the time required for puncture of the distal radial artery. In the present study, the time from local anesthesia to successful puncture for DRA averaged 5.3 min, compared to a shorter time of 2.9 min for SA. However, no significant differences were observed in the overall procedure time. This may be attributable to the fact that SA requires additional time for manual compression, whereas DRA allows for faster hemostasis. When accounting for the shorter hemostasis time, we believe that the slightly longer puncture time for DRA is clinically acceptable.

DRA is considered useful for VAIVT in patients with AVF, in whom access from the veins is difficult due to multiple lesions or poor vein maturation. In this study, the percentage of patients with early-phase VAIVT was significantly higher in the DRA group than in the SA group. A previous study reported that an AVF should ideally be left to mature for at least 14 days before the first cannulation [17]. It has also been reported that it will take a more extended period for AVF to develop to the point where they can be used in daily practice without problems. Huber et al. reported that the maturation rate of AVFs was 67% 6 months after creation, although almost one-third required an intervention to facilitate maturation or treat complications [3]. Considering this, many cases require VAIVT for veins that have not matured after AVF creation, and DRA has an advantage over venous access in these patients. In addition, stenosis with multiple lesions was significantly greater in the DRA group than in the SA group. This was likely the case because the operator judged treating multiple stenotic lesions using a venous approach challenging.

TRA is an approach similar to DRA and has been reported as an option in VAIVT procedures for AVF [8]. However, in conventional TRA situated on the palmar side of the wrist, sheath placement is often complicated in cases of forearm AVF because the puncture site is just above the anastomosis site. The DRA is positioned approximately 3–5 cm further distally which results in the securing of additional sheath positions.

One issue with catheterization is the diameter of the sheath that can be inserted into the access site. Naito et al. reported that the diameters of the radial and distal radial arteries assessed by ultrasound in men were 2.62 ± 0.60 mm and 2.04 ± 0.43 mm, respectively. In women, these diameters were 2.44 ± 0.51 mm and 1.96 ± 0.44 mm, respectively. They demonstrated that the distal radial artery is significantly smaller than the radial artery [18]. Thus, although the distal radial artery has a smaller diameter than the radial artery, it has also been reported that up to 6 Fr sheaths can be available. In the CONDITION study, a randomized clinical trial evaluating the effect of DRA on long-term radial artery occlusion, a 6 Fr artery sheath (RADIFOCUS INTRODUCER II, Terumo, Japan) was used in all DRA cases, and the success rate of the procedures was 100% per protocol [19]. There are significant advantages when using 6 Fr sheaths in DRA. The percentage of patients who underwent repeated interventions within 6 months after VAIVT was estimated to be approximately 50% [20, 21]. To improve this poor treatment outcome, drug-coated balloons (DCB) have been used recently to prevent restenosis after VAIVT, and their clinical efficacy has been demonstrated [22, 23]. The IN. PACT AV (Medtronic, Fridley, Minnesota, USA) requires a sheath size of 6 Fr; therefore, the ability to use a 6 FR sheaths when performing VAIVT by TRA is favorable because it allows the combination with DCB treatment.

While a poor outcome warrants the use of DCB, it also gives VAIVT a certain advantage. Thus, VAIVT often requires repeated treatment in many cases. The need for repeated treatment implies that access sites must be available repeatedly. In this study, 94.4% of the patients who underwent revascularization after an approach from the DRA could use the DRA again which indicates that DRA qualifies as a reusable access site.

### Safety of distal radial access for VAIVT

It has been reported that DRA is superior to radial access regarding the time required to achieve hemostasis after PCI [24]. In this study, there was no incidence of hematoma in the DRA group after VAIVT. DRA in VAIVT procedures was considered to provide safe hemostasis, similar to PCI.

Although the DRA approach is considered beneficial, a significant concern is the development of DASS due to distal radial artery occlusion after VAIVT procedures with DRA. In this study, no cases in the DRA group presented with symptoms of DASS; meanwhile, it has been reported that the incidence of distal radial occlusion was 3.5% during the 24-h follow-up period after PCI procedures [25]. DASS is a complication of AV access in patients undergoing hemodialysis. Asymptomatic steal has been reported to occur in up to 90% of dialysis access cases. In comparison, clinically significant DASS requiring intervention is seen in 0.3–2% of those with forearm access and 4–9% of those with upper arm access [26, 27]. Four vascular beds are involved in the cause of DASS: arteries proximal to the AV access, arteries distal to the AV access, arterial collateral vessels, and veins from the AV access [28]. While obstruction of the DRA can contribute to DASS, the vessels involved in the pathogenesis of DASS are complex. There have been reports of DASS treated with coil embolization of the distal radial artery in cases where blood flow from the side of the ulnar artery was steered into the shunt vessel, and DASS developed [29], suggesting that occlusion of the DRA does not necessarily lead to symptomatic DASS. In this study, among the 90 patients in the DRA group who required revascularization, the one patient with distal radial artery occlusion did not present with symptomatic DASS. In addition, none of the four patients with stenosis in the distal radial artery had symptomatic DASS. However, no patient in this study presented with DASS when VAIVT was performed; therefore, the safety of DRA in patients already presenting with symptoms of DASS remains unclear.

## Limitations

This study had certain limitations. First, this was a single-center, non-randomized, retrospective study, with access sites determined by the operator, indicating potential selection bias. Second, no patients in this study complained of DASS when VAIVT was performed; therefore, the safety of DRA in patients already presenting with symptoms of DASS remains unclear. Third, standard balloon-only treatment can be performed with small sheaths of less than 6 Fr. However, in this study, 6 Fr were always used to enable the use of DCB or scoring balloon. Fourth, this study included cases in which DRA was considered but changed from DRA to SA based on ultrasound findings. Prospective studies are warranted to reveal the success rate of intention-to-treat DRA.

## Conclusions

This study demonstrates that DRA is a feasible and safe strategy for VAIVT while also allowing repeated use; therefore, it can be considered a viable option for VAIVT.

## Supplementary Information

Below is the link to the electronic supplementary material.

Supplementary file1 (PDF 264 KB)

## Abbreviations

AV: Arteriovenous
AVF: Arteriovenous fistulas
DASS: Dialysis access-associated steal syndrome
DCB: Drug-coated balloons
DRA: Distal radial access
PCI: Percutaneous coronary intervention
SA: Standard access
TRA: Trans-radial approach
VAIVT: Vascular access intervention therapy
