# Supplementary material for: Feasibility and safety of the distal radial access for vascular access interventional therapy
Source: Cardiovasc Interv Ther. 2025 May 4;40(3):599–606. doi: 10.1007/s12928-025-01127-4 (PMC12167267; doi:10.1007/s12928-025-01127-4)
Supplement: Supplementary file 1 — Supplementary file1 (PDF 264 KB) [file 12928_2025_1127_MOESM1_ESM.pdf]

Online Resource

Feasibility and safety of the distal radial access for vascular access interventional therapy

Cardiovascular Intervention and Therapeutics

Short title: Kuroda K et al. Distal radial access for VAIVT

Authors:
Koji Kuroda, MD, PhD[1], Ayaka Murakami, MD[1], Takafumi Todoroki, MD, PhD[1], Masamichi Iwasaki, MD[1], Junichi Imanishi, MD, PhD[1], Souichiro Yamashita, MD, PhD[1], Wataru Fujimoto, MD, PhD[1], Makoto Takemoto, MD, PhD[1], Masanori Okuda, MD, PhD[1]

Affiliation: Department of Cardiology, Hyogo Prefectural Awaji Medical Center, Sumoto, Japan

E-mail address:
Koji Kuroda: k722black@yahoo.co.jp
Ayaka Murakami: bussan05151118@gmail.com
Takafumi Todoroki: todochan5028@gmail.com
Masamichi Iwasaki: iwa_michi1114@yahoo.co.jp
Junichi Imanishi: junnnichi@gmail.com
Souichiro Yamashita: soyamst@gmail.com
Wataru Fujimoto: watarutaru_328@yahoo.co.jp
Makoto Takemoto: t_makoto517@yahoo.co.jp
Masanori Okuda: okudamm11@gmail.com

Table of contents		Page
1	Online Resource 1	2
2	Online Resource 2	3

Online Resource 1. Comparison between circuit reconstruction cases and non-circuit reconstruction cases

Variables	Non-circuit reconstruction (n=413)	Circuit reconstruction (n=8)	P value
**Access cites**			0.69
DRA	177 (42.9)	4 (50.0)	
SA	236 (57.1)	4 (50.0)	
**AV circuit**			0.58
AVF	371 (89.8)	7 (87.5)	
AVG	42 (10.2)	1 (12.5)	
**Cite of AV circuit**			0.24
Forearm	328 (79.4)	5 (62.5)	
Upper arm	85 (20.6)	3 (37.5)	
**Culprit lesion**			
Occlusion	114 (27.6)	8 (100.0)	<0.001

Values are presented as mean ± SD or absolute numbers (%).

For continuous variables, the two groups were compared using a two-tailed, unpaired t-test or Wilcoxon test. Discrete variables are presented as percentages, and comparisons were performed using chi-square analysis or Fisher's exact test.

AV circuit: arteriovenous circuit; AVF: arteriovenous fistulas; AVG: arteriovenous graft; DRA: distal radial access; SA: standard access

Online Resource 2. The incidence of circuit reconstruction in subgroups

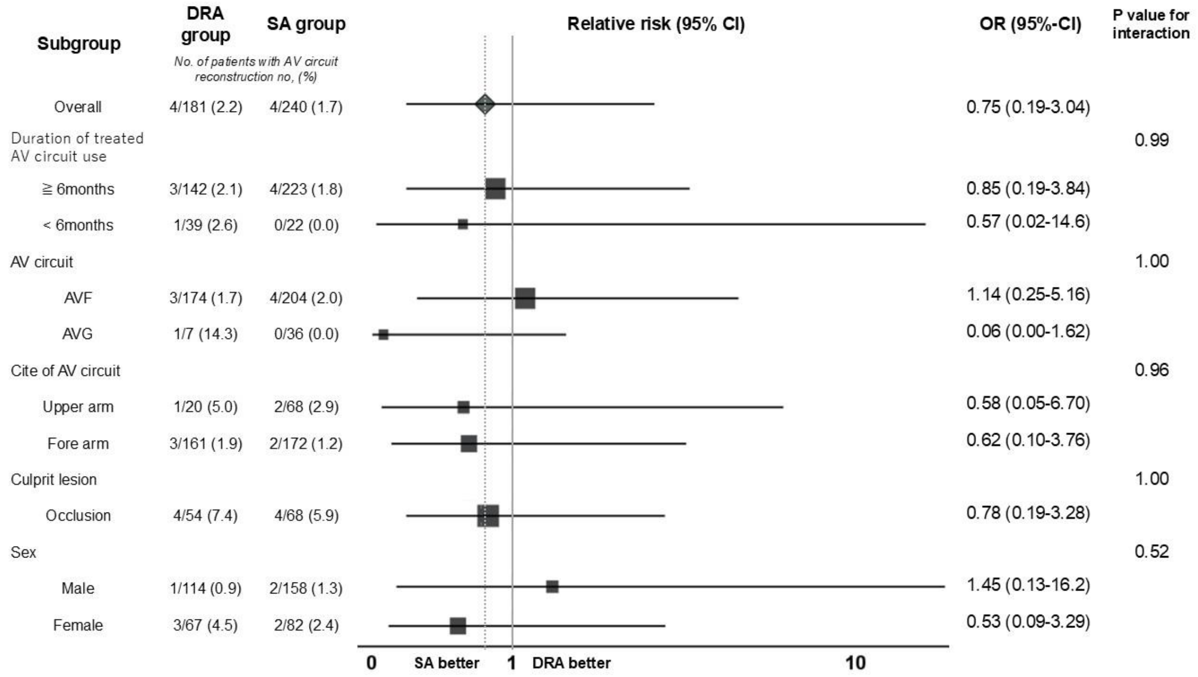

AV circuit: arteriovenous circuit; AVF: arteriovenous fistulas; AVG: arteriovenous graft; DRA: distal radial access; OR: odds ratio; SA: standard access

(Statistical Analysis) We conducted a multiple logistic regression analysis using SPSS version 29.0 (IBM Corp., Armonk, NY, USA). To examine the interaction effect between approach cite (X1) and variables (X2) on circuit reconstruction, an interaction term was created by multiplying the two independent variables. The model included X1, X2, and the interaction term (X1 × X2) as predictors. The p-value for interaction was derived from the Wald test in the "Variables in the Equation" table of the logistic regression output. A p-value < 0.05 was considered statistically significant.
